# An Atypical Presentation of a Male with Oral-Facial-Digital Syndrome Type 1 Related Ciliopathy

**DOI:** 10.1155/2016/3181676

**Published:** 2016-08-29

**Authors:** Sheena Sharma, Jennifer M. Kalish, Ethan M. Goldberg, Francis Jeshira Reynoso, Madhura Pradhan

**Affiliations:** ^1^Division of Nephrology, The Children's Hospital of Philadelphia, Philadelphia, PA, USA; ^2^Division of Human Genetics, The Children's Hospital of Philadelphia, Philadelphia, PA, USA; ^3^The Department of Pediatrics, The Perelman School of Medicine, The University of Pennsylvania, Philadelphia, PA, USA; ^4^Division of Neurology, The Children's Hospital of Philadelphia, Philadelphia, PA, USA; ^5^Department of Neurology, The Perelman School of Medicine, The University of Pennsylvania, Philadelphia, PA, USA

## Abstract

*Background*. Oral-facial-digital syndrome type 1 (OFD1) is a rare condition with X-linked dominant inheritance caused by mutations in the* Cxorf5 *(*OFD1*) gene. This gene encodes the OFD1 protein located within centrosomes and basal bodies of primary cilia. Approximately 15–50% of patients with OFD1 progress to end-stage kidney disease following development of polycystic changes within the kidneys. This condition almost always causes intrauterine lethality in males.* Description of Case Diagnosis and Treatment*. A Caucasian male aged 9 years and 9 months presented with increased urinary frequency, increased thirst, and decreased appetite. Physical examination demonstrated short stature, hearing loss, photophobia, murmur, and hypogonadism. He had no other dysmorphic features. Laboratory results revealed anemia, renal insufficiency, and dilute urine with microscopic hematuria but no proteinuria. Ultrasound showed small kidneys with increased echogenicity but no evidence of cystic changes. A Ciliopathy Panel showed a novel and likely pathogenic deletion, approximately 7.9 kb, in the* OFD1* gene encompassing exons 16, 17, and 19 (c.1654+833_2599+423del). Brain MRI did not demonstrate typical OFD1 findings. He is currently on chronic hemodialysis awaiting transplant from a living donor.* Conclusions*. We present a male patient with* OFD1* mutation who lacks the classic OFD1 phenotype who presented with end-stage renal disease without evidence of polycystic changes within the kidneys.

## 1. Introduction

Oral-facial-digital syndrome is a rare, X-linked dominant ciliopathy with multiorgan involvement. Currently 11 types of this syndrome exist and an additional 2 have been proposed. The incidence of OFD1 is 1/50,000–1/250,000 live births with no particular racial preference [1, 2]. It occurs as a result of missense, nonsense, frameshift, splicing, or gross deletions within the* OFD1* gene which is localized to a 12-Mb interval on* Xp22* [3]. More than 100 mutations have been identified [4]. OFD1 is X-linked dominant ciliopathy resulting in in utero demise in affected males, although sporadic mutations occur within 75% of female cases [1]. Mutations within* OFD1* are also responsible for 4 recessive X-linked phenotypes including mental retardation with macrocephaly, Simpson-Golabi-Behmel syndrome type 2, Joubert Syndrome type 10, and Retinitis Pigmentosa 23 [5].* OFD1* gene escapes X-inactivation, which means that it is expressed from both X chromosomes in females (while most genes are expressed from only one X chromosome) [6]. Approximately 15% of X-linked genes in humans escape X chromosome inactivation in humans [7]. Deletions lead to a truncated protein, which may result in a dominant negative effect on the normally produced protein. The levels of protein made from the active and the inactive (escaping) X chromosomes likely vary between individuals, and random X-inactivation results in phenotypic variability, even between individuals within the same family [8]. Balaton and Brown reported that DNA sequence, chromatin structure, and chromosome ultrastructure are all important factors in determining differences within escape genes [9].

This gene encodes OFD1 protein, which is localized to centrosomes and basal bodies of ciliated cells [10]. Patients with this condition have the characteristic facial features of hypertelorism, broad nasal root, and hypoplasia of the nasal alae; oral changes including cleft palate, hamartoma of the tongue, hypodontia, hyperplastic frenulum, and cleft or pseudocleft upper lip; and hand changes including brachydactyly, syndactyly, clinodactyly, and polydactyly [6]. Approximately 40% of patients have central nervous system involvement with structural and/or intellectual involvement [6]. Renal involvement consists of polycystic kidney disease (PKD). These cysts tend to arise primarily from the glomerulus and be irregularly distributed within renal parenchyma [11]. PKD affects approximately 15–50% of patients with OFD1 [12]. In a large cohort of 34 patients with OFD1, those without PKD had normal renal function [12]. To our knowledge, this is the first patient with OFD1 with absent classic oral, facial, or digital features and who presented with end-stage renal disease with absence of typical polycystic appearance on imaging.

## 2. Case Report

A male aged 9 years and 9 months presented to the Emergency Department for evaluation of a one-week history of increased urinary frequency and thirst. He also complained of decreased appetite and was noted to have a 7 lbs unintentional weight loss over the past week. Given his constellation of symptoms in the setting of a strong family history of diabetes mellitus, he was seen by his primary care physician earlier that day where a blood glucose finger stick was found to be elevated at 138 mg/dL and urinalysis showed glucosuria with 100 mg/dL. He was then referred to the Emergency Department for further care.

His birth history was remarkable for prematurity as his mother was induced at 36 weeks for intrauterine growth restriction. He was born via Cesarean section secondary to fetal distress. His birth weight was 2690 grams and his birth length was 48.3 cm. He was monitored in the neonatal intensive care unit for 24 hours for concerns of hypoglycemia given maternal gestational diabetes but his blood glucose levels remained within normal range. He was noted to have jaundice but did not require phototherapy. He passed his newborn hearing screen prior to discharge.

His past medical history was significant for an endocrine evaluation at 4 years and 3 months of age for polyuria, polydipsia, and weight gain. His weight was stable in the 50th percentile until approximately 2 years of age when it increased to the 90th percentile. His weight was in the 99th percentile at the time of his endocrinology clinic visit. His height had decreased from the 25th percentile at 3 years of age to approximately the 15th percentile at the time of his endocrinology visit. He underwent evaluation which was remarkable for an elevated fasting blood glucose level of 113 mg/dL and mildly elevated thyroid stimulating hormone at 5.3 mIU/L (normal range 0.5–4.3 mIU/L). His serum creatinine was 0.41 mg/dL and electrolytes were unremarkable. Further studies were recommended for evaluation of thyroid function and Cushing's syndrome but not completed at that time.

He also had a history of multiple sinus and ear infections requiring adenoidectomy and four sets of myringotomy tubes being placed over his lifetime. He had speech delay as sequela of his frequent ear infections and was receiving speech therapy twice weekly and hearing therapy three times weekly. He had received physical and occupational therapy services until he was in kindergarten for delayed milestones including walking at 16 months of age. He receives additional support for reading. Formal academic testing was recommended by his school which has not yet been completed. He had undergone an immunologic evaluation at an outside hospital due to his frequent infections which was unremarkable. He was diagnosed with asthma starting at 1 year of age and was prescribed fluticasone and cetirizine.

His family history was notable for maternal renal injury secondary to diabetes occurring during pregnancy but not requiring renal replacement therapy and photophobia in his mother, nephrolithiasis in the maternal grandfather and maternal aunt, hypertension in the maternal grandfather, and hearing loss and photophobia in a first cousin on the maternal side. There was no family history of consanguinity.

Review of systems revealed subjective chills and constipation. He also admitted to waking at night to drink and to void. He denied fever, abdominal pain, daytime or nighttime enuresis, urgency, dysuria, inability to empty his bladder, or hematuria.

In the Emergency Department his blood pressure was 123/71 mmHg, weight was 37 kg (82nd percentile), and height was 127.5 cm (3rd percentile). His physical examination was remarkable for a grade 2/6 systolic ejection murmur and hypogonadism. There were concerns for photophobia during his eye exam. There were no other dysmorphic features noted. His initial laboratory work-up was significant for elevated serum creatinine of 4.4 mg/dL, low serum calcium of 7.7 mg/dL (8.8–10.1 mg/dL), and slightly elevated serum phosphorus of 6.1 mg/dL (3.7–5.6 mg/dL). His serum intact parathyroid hormone level was elevated at 340 pg/mL (9–52 pg/mL). His complete blood count revealed normocytic anemia with serum hemoglobin of 8.1 g/dL (11.5–15.5 g/dL). His urinalysis showed moderate blood, no proteinuria, no leukocyte esterase, and a specific gravity of ≤1.005. His renal/bladder ultrasound showed small kidneys with right kidney 6.6 cm and left kidney 7.8 cm with increased echogenicity bilaterally and no evidence of cystic disease. He had an echocardiogram given his finding of a murmur which was unremarkable. An ophthalmology exam was performed with concern for cystinosis but cystine crystals were not visualized. He was diagnosed with bilateral retinal dystrophy with 20/50 OD and 20/30 OS. A bone age was obtained secondary to concerns of short stature which was greater than 2 standard deviations above his chronological age.

Given his constellation of symptoms and results of his evaluation, the suspicion for a ciliopathy, specifically juvenile nephronophthisis or Bardet-Biedl, was high.* ALMS1* gene testing for Alström syndrome and a commercial Ciliopathy Panel (from Prevention Genetics) were sent. The Ciliopathy Panel included 73 genes for disorders such as Joubert Syndrome, nephronophthisis, Bardet-Biedl, and Meckel syndrome. He had a computed tomography chest which was negative for bronchiectasis which was performed given his history of asthma and pulmonary system involvement with Alström syndrome.* ALMS1* gene testing was negative.

Genome-wide array analysis revealed a 3–12 Kb pathogenic hemizygous deletion within chromosome Xp22.2 that includes several exons of the OFD1 gene (OMIM # 300170, NM_003611). His Ciliopathy Panel showed a likely pathogenic deletion, approximately 7.9 kb, in the OFD1 gene encompassing exons 16, 17, and 19 (c.1654+833_2599+423del) which is a novel mutation. His mother underwent genetic testing and was found to have the same deletion. She lacks any characteristic features of OFD1.

He had a brain MRI which showed mild T2/fluid attenuation inversion recovery (FLAIR) hyperintensities in the medial temporal lobes bilaterally as well as a mildly thickened corpus callosum but no findings more typical of OFD1, such as intracerebral cysts, hydrocephalus, malformations of cortical development, agenesis of the corpus callosum, brainstem malformation, or Dandy-Walker malformation [13].

His renal function has continued to decline rapidly. Given symptomatic uremia, he has started on chronic hemodialysis. He is currently awaiting transplant from a living donor.

## 3. Discussion

Oral-facial-digital syndrome type 1 (OFD1) is a rare, X-linked dominant ciliopathy. Currently more than 100 mutations within the 23 exons of the* OFD1* gene on* Xp22* have been discovered [4]. These mutations include frameshift, missense, nonsense, splicing, or gross deletions [3]. The OFD1 protein, localized to the centrosome and basal body of primary cilia, is affected, resulting in cilia dysfunction [10].* OFD1* mutations affect the development of many tissues and organs including kidneys (metanephric mesenchyme), oral mucosa, tongue, nasal and cranial cartilage, limbs, and brain [11]. In addition, other features have been described including tremor, alopecia, osteoporosis, and potentially hearing impairment [14]. The classic oral, facial, and digital phenotype was absent in our patient. To our knowledge, this is the first patient reported with this condition who lacks any physical features typical of this disease and one of the few reported cases of an older male ever reported.

Renal involvement, consisting of polycystic kidney disease (PKD), has been noted in OFD1 [3]. Cystic changes can occur at any time point. Gillerot et al. described polycystic kidneys in a newborn male with OFD1 on autopsy performed shortly after birth [15]. Saal et al. examined a cohort of 34 females with OFD1. PKD was noted at a median of 29 years (2–52 years at diagnosis) and was found in 35% of the cohort [12]. A study by Prattichizzo et al. reported that renal cystic disease is present in 16% of patients less than 18 years of age and 63% of patients greater than 18 years of age [2]. Cysts are irregularly distributed and located within the cortex and the medulla. They are variable in size, multilocular, and thin-walled containing serous fluid [16]. These cysts tend to arise predominantly from the glomerulus and less from the distal tubule [17]. The kidneys are normal in size or palpably large but they maintain their reniform shape with minimal changes to renal contour [12]. Cystic changes in the liver and pancreas have also been described in OFD1 [10, 16]. Autosomal dominant and recessive polycystic kidney disease, tuberous sclerosis, and von Hippel-Lindau disease are all conditions in which cystic changes can appear early in life. ADPKD differs from OFD1 based on larger kidney size, distribution of cysts, tendency to larger cyst size, and origin of the cysts which are tubular in ADPKD. Our patient was found to have a pathogenic deletion in the* OFD1* gene encompassing exons 16, 17, and 19. There have been reports of cystic kidneys being more frequently associated with splice mutations and with mutations located in exons 9 and 12 on the* OFD1* gene [6, 18].

OFD1 patients with polycystic kidneys have a higher likelihood of developing renal failure. Saal et al. noted in their study that 10/12 patients with PKD had renal impairment with 9/12 having end-stage renal disease. They found that patients without PKD had normal renal function. The median age of end-stage renal disease in their cohort was 34 years [12]. However, end-stage renal disease has been reported ranging from 11 to 70 years in the literature [11]. Also, Toprak et al. mentioned that cystic changes are at times only discovered upon the diagnosis of renal failure [11]. To our knowledge, we present the first case of a patient with OFD1 diagnosed with end-stage renal disease with no evidence of PKD on imaging.

Our patient had a deletion mutation within the* OFD1* gene on Xp22. Mutations within this gene are responsible for several other phenotypes including Joubert Syndrome type 10, Simpson-Golabi-Behmel Syndrome type 2, and Retinitis Pigmentosa 23. Joubert Syndrome type 10 and Simpson-Golabi-Behmel Syndrome type 2 can both include cystic changes within the kidney, but Retinitis Pigmentosa involves only ophthalmologic changes with no other organ involvement. However, Joubert Syndrome type 10 and Simpson-Golabi-Behmel are associated with other phenotypic characteristics lacking in this patient and tend to be more severely affected or be associated with lethality in early infancy [19–21].

We present a male patient with novel* OFD1* mutation inherited in an X-linked dominant fashion. To our knowledge, this is the first patient reported with this condition who lacks typical phenotypic features including the presence of cystic kidneys who declined towards end-stage renal disease and one of the few older males ever reported. Mutations within this gene tend to cause intrauterine demise in males, and, as of the time of write-up, our patient is aged 10 years and 6 months. Based on this unusual presentation, we recommend that* OFD1* screening be considered, even in males, if the suspicion for ciliopathy remains high in families with features of OFD1 in female members.
